# A Brazilian cohort of individuals with Phelan-McDermid syndrome: genotype-phenotype correlation and identification of an atypical case

**DOI:** 10.1186/s11689-019-9273-1

**Published:** 2019-07-18

**Authors:** Claudia Ismania Samogy-Costa, Elisa Varella-Branco, Frederico Monfardini, Helen Ferraz, Rodrigo Ambrósio Fock, Ricardo Henrique Almeida Barbosa, André Luiz Santos Pessoa, Ana Beatriz Alvarez Perez, Naila Lourenço, Maria Vibranovski, Ana Krepischi, Carla Rosenberg, Maria Rita Passos-Bueno

**Affiliations:** 1 Centro de Pesquisa sobre o Genoma Humano e Células Tronco (CEGH-CEL), Instituto de Biociências, Universidade de São Paulo, São Paulo, Brazil; 2 Programa de Engenharia Química, Universidade Federal do Rio de Janeiro, Rio de Janeiro, Brazil; 3 Centro de Genética Médica, Universidade Federal de São Paulo (UNIFESP), São Paulo, Brazil; 4 Ambulatório de Neurogenética, Hospital Albert Sabin, São Paulo, Brazil; 5 Faculdade de Medicina, Universidade Estadual do Ceará, UECE, Fortaleza, Brazil

**Keywords:** Phelan-McDermid syndrome, 22q13.3 deletion syndrome, Autism spectrum disorder, *SHANK3*

## Abstract

**Background:**

Phelan-McDermid syndrome (PMS) is a rare genetic disorder characterized by global developmental delay, intellectual disability (ID), autism spectrum disorder (ASD), and mild dysmorphisms associated with several comorbidities caused by *SHANK3* loss-of-function mutations. Although *SHANK3* haploinsufficiency has been associated with the major neurological symptoms of PMS, it cannot explain the clinical variability seen among individuals. Our goals were to characterize a Brazilian cohort of PMS individuals, explore the genotype-phenotype correlation underlying this syndrome, and describe an atypical individual with mild phenotype.

**Methodology:**

A total of 34 PMS individuals were clinically and genetically evaluated. Data were obtained by a questionnaire answered by parents, and dysmorphic features were assessed via photographic evaluation. We analyzed 22q13.3 deletions and other potentially pathogenic copy number variants (CNVs) and also performed genotype-phenotype correlation analysis to determine whether comorbidities, speech status, and ASD correlate to deletion size. Finally, a Brazilian cohort of 829 ASD individuals and another independent cohort of 2297 ID individuals was used to determine the frequency of PMS in these disorders.

**Results:**

Our data showed that 21% (6/29) of the PMS individuals presented an additional rare CNV, which may contribute to clinical variability in PMS. Increased pain tolerance (80%), hypotonia (85%), and sparse eyebrows (80%) were prominent clinical features. An atypical case diagnosed with PMS at 18 years old and IQ within the normal range is here described. Among Brazilian ASD or ID individuals referred to CNV analyses, the frequency of 22q13.3 deletion was 0.6% (5/829) and 0.61% (15/2297), respectively. Finally, renal abnormalities, lymphedema, and language impairment were found to be positively associated with deletion sizes, and the minimum deletion to cause these abnormalities is here suggested.

**Conclusions:**

This is the first work describing a cohort of Brazilian individuals with PMS. Our results confirm the impact of 22q13 deletions on ASD and several comorbidities, such as hypotonia. The estimation of a minimal deletion size for developing lymphedema and renal problem can assist prediction of prognosis in PMS individuals, particularly those diagnosed in early infancy. We also identified one atypical individual carrying *SHANK3* deletion, suggesting that resilience to such mutations occurs. This case expands the clinical spectrum of variability in PMS and opens perspectives to identify protective mechanisms that can minimize the severity of this condition.

**Electronic supplementary material:**

The online version of this article (10.1186/s11689-019-9273-1) contains supplementary material, which is available to authorized users.

## Background

Phelan-McDermid syndrome (PMS) (OMIM #606232), or chromosome 22q13.3 deletion syndrome, is a rare genetic disorder that similarly affects males and females and shows considerable clinical heterogeneity [1]. PMS is characterized by developmental delay, absent or delayed speech, intellectual disability (ID), frequent autistic features, seizures, renal, cardiac and gastrointestinal abnormalities, and mild dysmorphisms [2, 3].

This syndrome results from *SHANK3* loss of function (or haploinsufficiency) mainly caused by deletions, whereas point mutations have also been described [2–4]. Deletion size varies from a few kilobases to more than 9 Mb, and frequently includes a 22q terminal deletion [3]. *SHANK3* encodes an abundant brain protein involved in regulating postsynaptic density of glutamatergic synapses, playing a role in synaptic function by modulating dendrite formation [5]. *SHANK3* loss of function is one of the most frequently recurring alterations in autism spectrum disorder (ASD) and ID cohorts, with an estimated frequency at 0.5% to 2%, respectively [1].

*SHANK3* haploinsufficiency is associated with the major neurological symptoms of PMS; however, it cannot explain the wide clinical variability among PMS cases [6] and recent data suggest that additional genes encompassed by the deletion also contribute to phenotype expressivity [7]**.** A number of genotype-phenotype correlation studies have been conducted, but there is no consensus among the results obtained [6, 8–11]. Further studies exploring the molecular basis of PMS are needed for clarifying the genetic basis for the clinical variability in PMS.

Therefore, our main goal was to explore the genotype-phenotype correlation in a cohort of 34 Brazilian individuals with PMS, representing an ethnic admixture of European, African, and Amerindian ancestries. We also estimated the frequency of PMS among Brazilian ASD individuals and in an independent Brazilian cohort of individuals with ID (associated or not with other clinical signs). This work expands the existing literature on PMS in different ethnicities, aiding in uncovering the etiology of its clinical variability.

## Methods

### Cohort

Thirty-four individuals with clinical and genetic diagnosis of PMS, members of the “Associação dos Amigos e Familiares da Síndrome de Phelan-McDermid - Brasil” (https://www.phelanmcdermidbrasil.com/), were included in this study. This cohort consisted of 16 males (47%) and 18 females (53%), with age ranging from 1.8 to 19.6 years old (mean = 7.54, SD = 3.86). Parents or guardians provided genetic exams and completed a standardized medical history questionnaire. The questionnaire included queries about the individuals’ development, comorbidities, autism diagnosis, and genetic tests. In addition to that, 27 families also provided pictures of their affected children.

Two other independent cohorts were included in this study in order to estimate the frequency of 22q13.3 deletions in Brazilian ASD or ID individuals. An ASD cohort of 829 individuals [656 (79.1%) males and 173 (20.9%) females] was tested by multiplex ligation-dependent probe amplification (MLPA) and ascertained at CEGH-CEL (Centro de Pesquisas sobre o Genoma Humano e Células Tronco, Instituto de Biociências (IB), USP) between 2009 and 2017, with a diagnosis or diagnostic hypothesis of ASD. The other cohort consisted of 2297 individuals, mostly under 18 years old and were referred to chromosomal microarray testing for presenting ID, either associated or not with other clinical signs. These samples were tested by the Laboratório de Pesquisa em Genética Humana from the Departamento de Genética e Biologia Evolutiva, IB, USP, from 2004 to 2018.

Parents or guardians of individuals from both cohorts signed a consent form. The study was approved by the Brazilian National Research Ethics Commission.

### Clinical evaluation

#### Dysmorphology and comorbidity evaluation

Morphological and comorbidity evaluation was performed by analyzing the individuals’ medical records and pictures, whenever available. Parents were asked to answer questions about comorbidities with three possible answers: “Yes” (if the individual has the comorbidity), “No” (if the individual does not have the comorbidity), and “I am not sure” (if the parents cannot answer). In some cases, such as “speech,” the answer: “not applicable” for individuals aged less than three was taken into account in our analyses. Any dubious or “not sure” answer was excluded. Moreover, three clinicians from the Departamento de Morfologia e Genética, Escola Paulista de Medicina, UNIFESP, independently evaluated pictures of the individuals in order to analyze their morphological features.

### Genetic tests to detect copy number variation

#### Molecular diagnosis of the 34 PMS individuals

All individuals have a deletion at 22q13.3 encompassing *SHANK3*. Different exams and platforms were applied: array-CGH (*n* = 15) or SNP array (*n* = 14) were performed in 29 individuals (see details in Table 1); fluorescent in situ hybridization (FISH) was performed in four individuals, and MLPA (SALSA MLPA kit P036-E2-0413 and P070-B3-0714 MRC Holland) was performed in one individual. All genetic tests were conducted by outsourced laboratories.

**Table 1:** Details of the 22q13.3 deletions identified in 34 individuals with Phelan-McDermid syndrome

ID	Gender	Copy number evaluation method	Rearrangement	Array coordinates (hg19[GRCh37])	Deletion size (Mb)	Genes	Inheritance
P1	F	SNP array^a^	Deletion	48810119–51211393	2.4	Many	NA
P2	F	aCGH 180K	Deletion	43371148–51186249	7.8	Many	NA
P3	F	FISH	Deletion	NA	NA	*SHANK3*	NA
P4	M	aCGH 180K	Deletion (mosaic)	42839080–51174293	8.3	Many	NA
P5	M	FISH	Deletion	NA	NA	*SHANK3*	NA
P6	M	aCGH 60K	Deletion	42865291–51219009	8.3	Many	De novo
P7	F	aCGH 180K	Deletion	49608334–51186249	1.5	Many	NA
P8	F	SNP array 750K	Deletion	46276401–51197766	4.9	Many	NA
P9	F	SNP array 400K	Deletion	48771374–51171678	2.4	Many	De novo
P10	F	aCGH^a^	Deletion	48434307–51178213	2.7	Many	De novo
P11	F	SNP array 400K	Deletion	50667787–51171678	0.5	Many	De novo
P12	M	SNP array 400K	Deletion	51122360–51171678	0.049	*SHANK3*	De novo
P13	M	SNP array 750K	Deletion	47557877–51197766	3.6	Many	NA
P14	M	aCGH^a^	Deletion	50241089–51178213	0.9	Many	NA
P15	M	SNP array 400K	Deletion	43492638–51197766	7.7	Many	NA
P16	M	SNP array 400K	Deletion	45235285–51171678	5.9	Many	De novo
P17	F	FISH	Deletion	51104247–51178574	0.074	*SHANK3*, *ACR*	NA
P18	F	aCGH 180K	Deletion	51123491–51224252	0.1	*SHANK3, ACR, RABL2B*	NA
P19	F	aCGH 44K	Deletion	49595567–51178405	1.5	Many	De novo
P20	F	aCGH 180K	Deletion	43213659–51224252	8.0	Many	NA
P21	F	SNP array 750K	Deletion	46168628–51115526	4.9	Many	NA
P22	M	MLPA	Deletion	NA	NA	*ARSA*, *SHANK3*	NA
P23	F	MLPA/FISH	Deletion	NA	NA	*SHANK3*	NA
P24	F	aCGH 60 K	Deletion	43213481–51178354	7.9	Many	NA
P25	F	SNP array 750K	Deletion (mosaic)	42399686–51197766	8.79	Many	De novo
P26	M	aCGH 180K	Deletion	49123097–51224252	2.1	Many	NA
P27	M	SNP array 850K	Deletion	42152988–51211392	9.0	Many	NA
P28	F	SNP array 400K	Deletion	47497833–51171678	3.67	Many	De novo
P29	M	FISH	Deletion	NA	NA	*SHANK3*	NA
P30	M	aCGH 180K	Deletion	47963467–51219009	3.25	Many	NA
P31	M	SNP array 750K	Deletion	50274217–51197766	0.9	Many	NA
P32	M	aCGH 180K	Deletion	51123491–51224252	0.1	*SHANK3, ACR, RABL2B*	NA
P33	F	SNP array 850K	Deletion	46814671–51211392	4.39	Many	NA
P34	M	SNP array^a^	Deletion	43094876–51197838	8.1	Many	NA

*NA* not available

^a^The outsourced laboratory did not specify the platform on the report

#### Multiplex ligation-dependent probe amplification (MLPA) test in 829 ASD individuals

The SALSA MLPA kit P343 AUT (Cat. P343-100R, MRC Holland, Amsterdam, the Netherlands) was used, which may detect alterations in 15q11-q13, 16p11.2, and 22q13.3. The procedure was performed as previously reported [12]. The PCR products were detected by the ABI 3130 Genetic Analyzer (Applied Biosystems, Foster City, CA, US), by using capillary electrophoresis and GeneMarker (Softgenetics) to analyze the raw data.

#### Array-CGH in the 2297 ID individuals

DNA sample of all individuals were submitted to CMA, by using different platforms, namely aCGH 60K and 180K, from Agilent Technologies (average probe spacing of 54.5 Kb and 17.6 Kb, respectively) as previously described [13].

### CNV pathogenicity interpretation

Every copy number variant (CNV) was characterized by type (deletion or duplication), size, gene content (based on RefSeq gene data available on the UCSC genome browser, using the hg19), allele population frequency (based on the Database of Genomic Variation Database - DGV), literature, and clinical information about disease-associated genes and inheritance (whenever possible). This information together with the International Standard Cytogenomic Array (ISCA) and the American College of Medical Genetic (ACMG) standards [14] were taken into account to classify the CNVs into pathogenic, benign, or variant of uncertain clinical significance (VOUS).

### ASD assessment

In total, 18 out of 34 individuals were evaluated by a specialist (neurologist, psychiatrist, or psychologist) for ASD. In addition to medical diagnosis, parents were asked to answer about autistic-like behavior or ASD, which considered the following autism symptoms: no-eye contact, repetitive behavior, lack of social interaction, difficulty in changing routines. The questionnaire presented four possible answers: (1) No, he/she has no autism symptoms; (2) No, a detailed assessment was made and autism was ruled out; (3) Yes, autism was diagnosed in an evaluation with a neurologist/psychologist/psychiatrist; (4) Yes, there has been no diagnosis but we know from his/her behavior.

### Data analysis

#### Non-parametric test

Descriptive statistics were calculated across all measures into three different domains: comorbidities, ASD, and language status. Mann–Whitney *U* tests were conducted to explore potential associations between each medical comorbidity and ASD with deletion size. Kruskal–Wallis was performed to determine whether there was a correlation between language status and deletion size. The level of significance adopted was *P* < 0.05.

#### Cluster analysis

Ward’s Hierarchical cluster analysis was performed using only the comorbidity variables in order to verify if individuals group according to their clinical features.

## Results

### Characterization of 22q13.3 deletions in PMS individuals

**Fig. 1:**
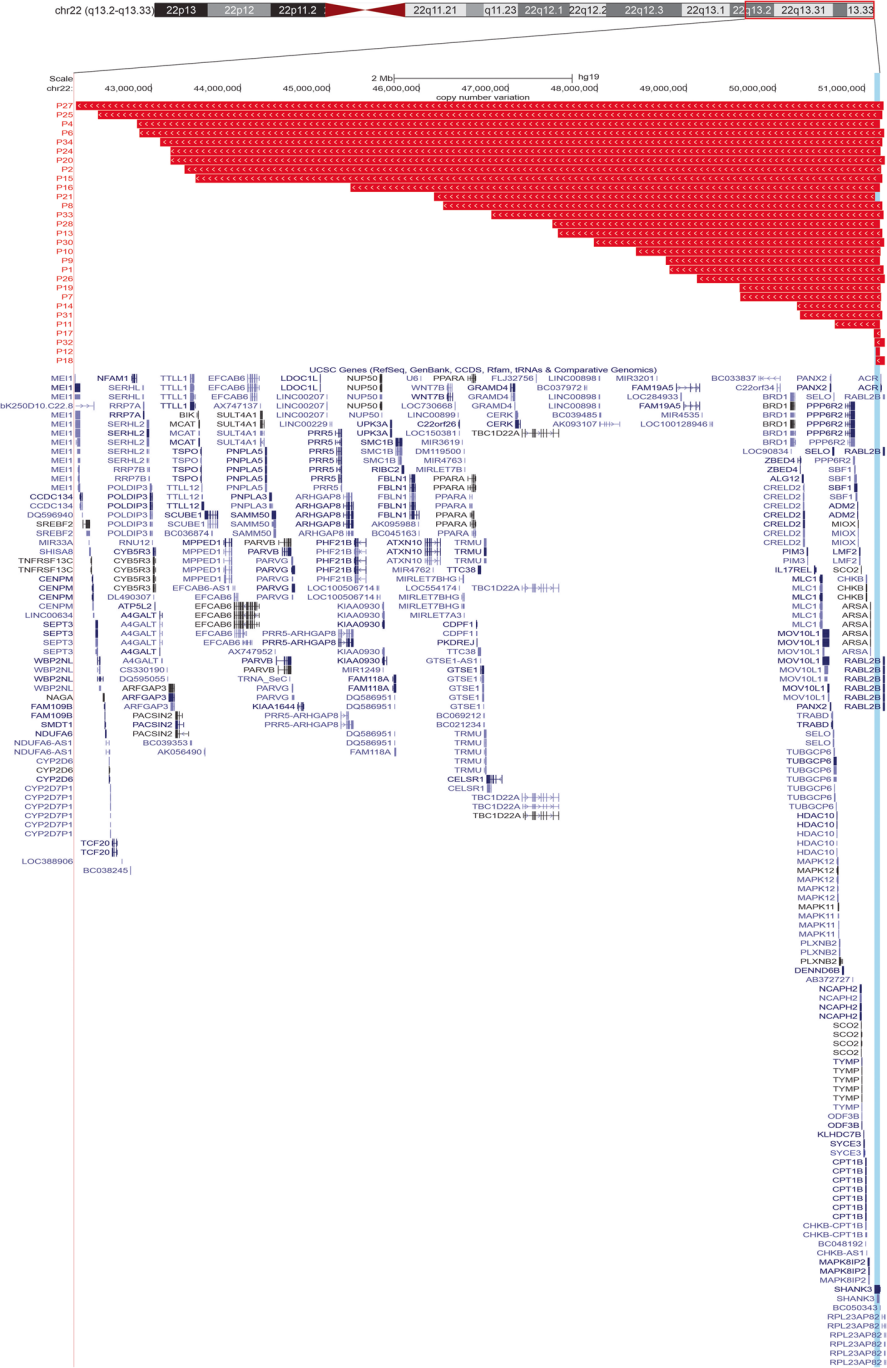
Distribution of terminal deletions among 29 PMS individuals that varied from 49 kb to 9.1 Mb

All individuals carry deletions affecting *SHANK3* (Table 1). Deletion size was estimated in 29 individuals tested by CMA; they carried terminal deletions with a mean size of 4.1 Mb (ranging from 0.049 to 9.1 Mb) (Fig. 1 and Table 1). Two of them showed mosaicism: one with an 8.3 Mb deletion (P4) and the other with an 8.8 Mb deletion (P25). Parental genetic testing was available for nine individuals and all deletions were de novo.

### Other genetic alterations in the Brazilian PMS cohort

We also identified additional rare CNVs (*n* = 10) located in genomic regions other than 22q13.3 in six individuals (Table 2). Three out of six individuals carried terminal duplications varying in size from 0.4 to 14.9 Mb, thus suggesting that the genomic imbalances resulted from the presence of a translocation derivative chromosome. The remaining three individuals had interstitial CNVs varying in size from 0.03 to 14.8 Mb. All these additional CNVs encompass OMIM genes. Of these, two are associated with genomic alterations that clinically overlap PMS: 12q24 duplication (P4) and chromosome 16p13.3 duplication syndrome (P31); therefore, they were classified as pathogenic. Moreover, one CNV (P28) affects a gene associated with autosomal dominant non-insulin-dependent diabetes mellitus. It was not possible to establish whether the remaining CNVs added to the clinical variability of these individuals, since they encompass regions with genes that were either not previously associated with disorders or associated with recessive disorders; therefore, they were classified as variants of unknown clinical significance (VOUS).

**Table 2:** Non-22q CNV findings in nine individuals with PMS

ID	Additional genomic findings (hg19[GRCh37])	Size	Position	Genes	OMIM disease association
P4	Yq11.21q11.23 (13872502-28644194)×0	14.8 Mb	Interstitial	32 protein-coding genes (USP9Y)	Spermatogenic failure, Y-linked, 2 SPGFY2 and Chromosome Yq11 interstitial deletion syndrome
	12q24.23q24.33 (118841028-133773528)×3 (mosaic)	14.9 Mb	Terminal	103 protein-coding genes (18 morbid)	Many
P12	Xq28(148685454-148716519)×3	31 kb	Interstitial	*TMEM185A*	No OMIM disease association
P20	18p11.32 (64847-464868)×3	400 kb	Terminal	*USP14*, *THOC1*, *COLEC12*	No OMIM disease association
	Xq28(148094899-148607966)×3	513 kb	Interstitial	*IDS*	Mucopolysaccharidosis II (XLR^a^)
P21	22q13.31 (44257185-45143609)×3 (mosaic)	886 kb	Interstitial	8 protein-coding genes (none morbid)	No OMIM disease association
	Xp22.33 (7514750-8135644)×3	620.9 kb	Interstitial	*VCX, PNPLA4*, *MIR651*	No OMIM disease association
P28	15q21.3 (58801559-58861468)×1	59.9 kb	Interstitial	*LIPC*	Hepatic lipase deficiency (AR^b^), [High density lipoprotein cholesterol level QTL 12], {Diabetes mellitus, non-insulin-dependent} (AD^c^)
P31	11p14.3 (23032300-24843680)×3	1.8 Mb	Interstitial	*LUZP2*, *MIR8054*	No OMIM disease association
	16p13.3 (85880-3998442)×3	3.9 Mb	Terminal	158 protein-coding genes (28 morbid)	Chromosome 16p13.3 duplication syndrome

^a^X-linked recessive inheritance

^b^Autosomal recessive inheritance

^c^Autosomal dominant inheritance

### Clinical findings in Brazilian cohort of PMS individuals

Several dysmorphisms were evaluated in 26 individuals (Additional file 1: Table S1). The most common morphological features (frequency > 70%) were ear abnormalities, sparse eyebrows, large/fleshy hands, and fine fingers. Regarding comorbidities, the most commonly reported were increased pain tolerance and hypotonia, occurring in 80% and 85% of the individuals, respectively (Table 3, Additional file 1: Table S2). In addition, 60% of individuals had a history of recurrent upper respiratory tract infections (Table 3). Other common conditions (frequency > 50%) were gastroesophageal reflux disease and chewing difficulties (Table 3).

**Table 3:** Reported comorbidities in individuals with PMS in our cohort as compared to the literature

Comorbidity	*N*	Total	Present study	Literature frequency^a^
Hypotonia	28	33	85%	29–100%
Increased pain tolerance	24	30	80%	10–88%
Recurring upper respiratory tract infections	20	33	61%	8–53%
Chewing difficulties	19	33	58%	50%^b^
Gastroesophageal reflux	17	30	57%	> 25–44%
Constipation and/or diarrhea	14	33	42%	38–41%
Sleep disturbance	13	33	39%	41–46%
Renal abnormalities	10	33	30%	17–38%
Seizures (febrile and/or non-febrile)	9	34	26%	14–41%
Precocious or delayed puberty	4	28	14%	0–12%
Lymphedema	4	30	13%	22–29%
Cardiac abnormalities	2	34	6%	3–25%
Hearing loss	1	34	3%	NA^c^

^a^Frequencies based on the literature review available [15]

^b^Frequency available in the analysis of PMS individuals carrying *SHANK3* point mutations [16]

^c^Although the frequency of this comorbidity was not considered in this study, the literature describes some cases of PMS individuals presenting hearing loss [17, 18]

### Development

All except one individual included in this study have neurodevelopmental delay. We evaluated language and motor development based on the information the parents reported. For language status, we considered only individuals above 3 years old, who were classified into four different classes: speech absence, unintelligible speech (babbling), speaks a few words, and speak sentences. Eighty-four percent (21/25) of children showed unintelligible or absent speech, and only three children (3/25; 12%) speak sentences (P14, P30, and P32) and were respectively 9, 11, and 19 years old at the time of evaluation. Regarding motor development, 75% (25/33) of them walk, with a mean age of onset at 2.27 years old (SD = 0.84). The remaining eight individuals did not walk at the time of evaluation (aged from 1.8 to 10).

Furthermore, individual P32 has a phenotype that differs from that of the other PMS individuals and is described in detail below.

### Atypical individual in the Brazilian PMS cohort

Individual P32 is a 20-year-old male diagnosed with PMS at the age of 18, when he failed a psychometric test when applying for a driver’s license. His karyotype was normal. Array-CGH analysis revealed the deletion of 110 kb in one allele in 22q13.3, that partially encompasses *SHANK3* (from exon 10 toward its 3′ end) and two other genes (*ACR* and *RABL2B*). The MLPA test in his parents confirmed that the CNV in P32 individual is a de novo mutation (Additional file 1: Figure S1). He is the only child of healthy non-consanguineous parents; her mother gave birth at the age of 26 by vaginal delivery at 40 weeks of gestation (Apgar scores 8/9, weight 3650 g, length 52 cm). Early development was characterized by neonatal hypotonia, and he had head control at 8 months of age. He started speaking at the age of 1 and walked when he was 1 year and 8 months old. Literacy occurred on a regular basis and he attended regular elementary through high school programs, without the need for additional assistance. At the age of 20, Wechsler Adult Intelligence Scale showed verbal, performance, and full scale IQ at 93, 99, and 96, respectively. The individual presents deficits in attention and in processing speed, in addition to difficulty in adjusting to social norms. He also presents mild phonemic impairment and some autistic-like features, such as difficulties related to making and keeping friendships, social and emotional responsiveness, processing figured speech, sensory sensitivity, and the insistence on sticking to routine. In turn, executive functions, such as logical reasoning, abstraction, and operational memory, are preserved. Currently, he is in the seventh semester of studies toward getting his bachelor’s degree in Computer Sciences at a private university. Clinical evaluation at the age of 20 showed dysmorphic features, such as elongated face, hypertelorism, prognathism, small and slightly bulbous nose, and arched palate. Doppler echocardiography, electroneurography, magnetic resonance imaging (RM) and electroencephalogram (EEG), abdominal and renal ultrasound, and audiological evaluation were performed between 2015 and 2018 and showed normal results.

### ASD in PMS

Furthermore, 73.5% (25/34) of individuals presented autism. ASD was diagnosed by a specialist in 18 of them (ASD frequency: 13/18 or 72.2%), while the remaining 16 had the ASD diagnosis based on the questionnaire (ASD frequency: 12/16 or 75%)

### Frequency of *SHANK3* copy number alterations in ASD and in other neurodevelopmental disorders

In a Brazilian cohort of 829 individuals with ASD diagnosis, we observed that five of them (all males) presented a 22q13.3 deletion (0.60%). In another cohort consisting of 2297 ID individuals, 15 of them presented *SHANK3* deletions (0.61%).

### Genotype-phenotype correlation

**Fig. 2:**
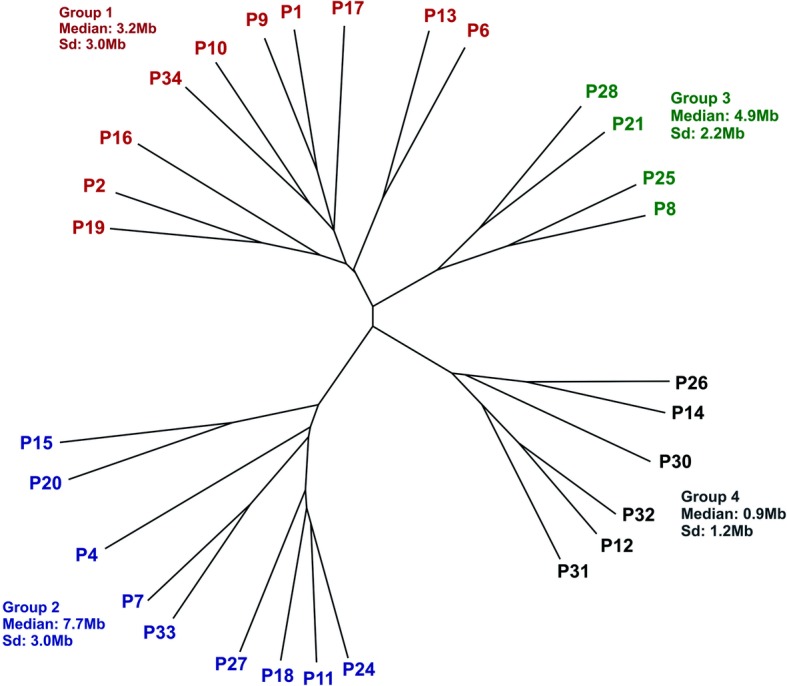
Hierarchical clustering dendrogram graphic showing the formation of four clusters of individuals according to their comorbidities. The range of deletion size is summarized for each group. *Sd* standard deviation

Twenty-nine individuals with CMA results were included for genotype-phenotype correlation analysis. A cluster hierarchical analysis was performed in order to determine similarities among groups of PMS individuals using comorbidity data and resulted in four groups that mainly differ in frequency and type of comorbidities (Fig. 2; Additional file 1: Table S3). In this analysis, the degree of severity of each comorbidity was not considered. A positive relationship between the groups and the median of deletion size of their members was observed, but with a borderline *P* value (*P* = 0.05, Kruskal–Wallis test). Groups 2 and 4 presented the largest and smallest median deletion size, respectively. Group 4 consisted of individuals who were characterized by the absence of some comorbidities, such as lymphedema, renal abnormalities, gastroesophageal reflux, strabismus and sleep disturbance, which are clinical features that are present in some individuals from group 2. Once we tested the correlation between each comorbidity individually with the deletion size (Mann–Whitney test), only renal abnormalities (*P* value = 0.011) and lymphedema (*P* value = 0.0480) had a significant correlation (Additional file 1: Table S4). The two individuals with additional pathogenic CNVs (P4 and P31) were clustered in the expected group according to their 22q13 deletion size, that is, P4 (deletion of 8.3 Mb) clustered to group 2 (largest deletion) and P31 (0.9 Mb) to group 4 (smallest median deletion).

We also tested the correlation of deletion size with language status and autistic features. The only three individuals that were able to speak full sentences (P14, P30, and P32) were in the group with the smallest mean deletion size (group 4), thus suggesting a correlation with deletion size, which was statistically confirmed (Kruskal–Wallis, *P* = 0.04). Finally, we did not find positive correlation between ASD and deletion size (Mann Whitney, *P* = 0.38), nor between groups from the clustering analysis and ASD frequency (Chi-square = 0.2651).

In order to verify the smallest regions in 22q13.3 that were more likely associated with renal abnormalities, we analyzed 14 individuals from the literature [9, 13, 14] with deletions in the 22q13.3 region detected by CMA who presented renal abnormalities (Additional file 1: Figure S2 and Table S5). The smallest deletion found was 1.3 Mb, as described by Tabet et al. (2017). We also determined the penetrance of renal abnormalities in individuals with deletions encompassing four candidate genes for renal abnormalities: *ZBED4*, *CELSR1*, *FBLN1*, and *UPK3A*, by using data from 101 individuals *(*72 individuals from the literature in addition to our 29 individuals; Additional file 1: Table S6). The penetrance observed for alterations encompassing only *ZBED4*, the closest gene to *SHANK3*, was 14.7%, while for the alterations encompassing *ZBED4* and *CELSR1* was 36.4%, and for alterations encompassing all four genes was 40.0%.

## Discussion

The present study conducted in 34 Brazilian PMS individuals confirmed the great heterogeneity in deletion size at 22q13.3 encompassing *SHANK3* and the relevance of de novo mutations to the origin of these deletions [3]. None of our patients harbor pathogenic point mutations in *SHANK3*, a less frequent cause of PMS [16] detectable through sequence analysis, possibly due to misdiagnosis, as CNV analysis (by karyotype, MLPA, and CMA) is the most accessible genomic test for our population.

Ten CNVs in addition to the 22q13.3 deletion were observed in six out of 29 individuals (21%). Three of them (10%) carry a terminal duplication, in accordance to previous estimates [8, 15] and possibly represent cases where one of the parents harbors a balanced chromosomal translocation. Among the other non-22q13.3 genetic alterations, the 16p13.3 duplication is associated with facial dysmorphisms, language impairment, delayed motor development, and broad toes [2, 19, 20], which are clinical features also present in our individual (P31). The 12q duplication leads to clinical features commonly seen in PMS, such as growth retardation, neurodevelopmental delay, and dysmorphic features [21], and it may contribute to the severity of the phenotype of the PMS individual (P4). It is of note that these two individuals were clustered as expected by their *SHANK3* deletion size. Given the clinical overlap among these three genetic alterations, these results are expected, particularly since we only considered the presence/absence of comorbidities and not their severity in the cluster analysis. Nevertheless, it is possible that these additional non-22q13.3 hits have contributed to phenotype severity. Among the remaining non-22q13.3 CNVs, we would like to highlight an intragenic deletion encompassing *LIPC*, in which pathogenic variants lead to an autosomal dominant form of non-insulin-dependent diabetes with late onset (OMIM#151670). This is not a common clinical feature of PMS individuals [22], but this incidental finding could contribute to better manage the individual in adulthood.

The most common comorbidities in our cohort were increased pain tolerance and hypotonia, as previously reported by others [10]. We observed a high frequency of chewing difficulties (58% of the individuals), which have been previously reported in the literature [3, 10, 23]. We also observed a high frequency of sparse eyebrows in our cohort (80%), which is a feature described recently in two individuals with *SHANK3* point mutations [16] and it could be included as an additional clinical feature in the individuals’ morphological evaluation. Only three children were able to speak full sentences in our cohort, and about 75% of our individuals were able to walk but with a delayed onset, which is in line with a global developmental delay observed in different PMS cohorts [8].

Individual P32 represents an exception in PMS cohort, since he was only diagnosed at 18 years of age. He carries the smallest deletion involving *SHANK3* and no other pathogenic CNV was identified. He presents normal literacy skills, developed speech with slightly impaired phonemic fluency, and average IQ score (IQ = 96). To our knowledge, this represents the first case of *SHANK3* associated with normal IQ. Tabet et al. 2017 reported a mother of a individual of PMS with deletion 0.067 kb in *SHANK3*, who presents non-verbal IQ = 75, with no history of speech delay, no significant autistic symptoms, no psychiatric axis comorbidities, and normal medical history. Therefore, these cases expand the spectrum of clinical variability caused by haploinsufficiency of *SHANK3*. Deeper genomic studies in unusual cases, such as the one here reported, could unravel pathways and mechanisms that lead to a less severe clinical outcome. This approach could open new opportunities for drug discovery.

We observed a high frequency of ASD (~ 73.5%) in our cohort, confirming it as a clinical feature of PMS [6, 8–10, 16]. Although the presence of autistic features was surveyed via questionnaire in about half of the samples, the frequency of ASD was similar between those diagnosed by a medical professional and those whose autistic behavior was based only on the questionnaire. We did not observe significant correlation between ASD and deletion size, which can be attributed to limitations in sample size or in evaluating ASD in patients with severe neurodevelopmental delay generally associated with larger deletions [6, 8, 24]. Notably, even though we were not able to assess the degree of intellectual disability in most of our individuals as our method was questionnaire-based, all except one individual did have general developmental delay such as poor or lack of language and reduced cognitive ability, which is the main phenotype of PMS.

PMS is a relatively common syndrome among both individuals with ASD and intellectual deficiency [8, 16]. Indeed, we observed a PMS frequency at about 0.60% in both our ASD individuals and ID cohort. While the proportion of PMS in our ASD individuals was comparable to other estimates in the literature (~ 1%) [1, 25, 26], the frequency in ID fell within the lower range of those estimates (~ 2%) [25], possibly because ID cohorts are clinically more heterogeneous than those that include only ASD individuals.

The cluster analysis was performed to verify which individuals were more clinically similar to each other and which comorbidities discriminate the clusters. Unlike previous studies [6], our clustering included a larger number of comorbidities, resulting in four groups. Due to our relative small sample size, we opted to discuss only the findings with significant differences. Two out of these four groups differed from each other regarding to lymphedema, renal abnormalities, language impairment, which in turn were correlated with deletion size, reinforcing the association of these comorbidities with deletion size [6, 9, 10]. It is of note that lymphedema was only present in individuals with deletions larger than 4.3 Mb, and its onset occurred mainly after 10 years of age (three out four individuals), reinforcing that lymphedema is dependent on age [9]. Renal abnormalities were present in individuals with deletion larger than 1.3 Mb and this comorbidity was present in all clusters except for cluster group 4 (with lower mean deletion size), further corroborating that *SHANK3* disruption does not seem to be sufficient to cause renal abnormalities [16]. The penetrance of renal abnormalities in PMS individuals varies from 17 to 38% in the literature and at least four genes, *UPK3A*, *ZBED4*, *CELSR1*, and *FBLN1*, have been considered as candidates for this comorbidity [7, 11]. In our analysis, we observed that the penetrance of renal abnormalities was about 2.5 times higher in deletions involving *ZEBD4* and *CELSR1* than in deletion of *ZEBD4* only, thus suggesting that the development of renal abnormalities in PMS individuals could be caused by an additive effect of these genes. As the penetrance was only up to 40.0% when deletions involved all four candidate genes, it is likely that other factors contribute to this comorbidity in PMS.

## Conclusions

In summary, our data support the importance of genetic factors in the etiology of the clinical variability and confirm the findings regarding the impact of 22q13 deletions on several features such as ASD and hypotonia, and defines sparse eyebrows as a clinical dysmorphic feature in the Brazilian PMS cohort. We also showed that 6.9% of PMS individuals from our cohort have additional CNVs, including pathogenic ones, and we recommend that they be interpreted as being able to contribute to the phenotype. We estimated a minimal deletion size for the development of comorbidities such as lymphedema, renal problem, and speech impairment, an information that may change prognosis in PMS diagnosed in early life. We also identified the first case of a male individual carrying *SHANK3* deletion with a very mild phenotype, which reinforces the idea that some individuals show some degree of resilience to such mutations.

## Additional file

Additional file 1: **Figure S1.** Multiplex ligation-dependent probe amplification (MLPA) test of atypical individual (P32) and his parents, confirm that its a de novo deletion. **Figure S2.** 22q13 deletions in 22 patients with renal abnormalities. **Table S1.** Frequency of dysmorphic features seen in PMS individuals. **Table S2.** Clinical findings in Brazilian PMS individuals. N/A = not available; + = comorbidity is present in the individual; − = comorbidity absent in the individual; F = female, M = male. **Table S3.** Clustering analysis of PMS individuals according to the sex and comorbidities seen among them. **Table S4.** *p*-value of Mann Whitney test performed for each comorbidity’s frequency and deletion size in our cohort (*N* = 34). **Table S5.** Fourteen individuals with 22q13.3 deletion and renal abnormalities previously described in the literature for which data was available online. **Table S6.** One hundred and one patients from three previously published works (Tabet et al, 2017; Lei et al, 2016; Soorya et al, 2013) and from our cohort included in the analysis of the frequency of renal abnormalities. All chromosomal coordinates are based on the hg19 version of the human reference genome. Color blue = individuals from Tabet et al (2017), green = individuals from Soorya et al (2013), orange = individuals from our cohort, and black = individuals from Lei et al (2016). (DOCX 400 kb)

## Abbreviations

Array-CGH: Microarray-based comparative genome hybridization
ASD: Autism spectrum disorder
CMA: Chromosome microarray analysis
CNV: Copy number variant
FISH: Fluorescent in situ hybridization
ID: Intellectual disability
MLPA: Multiplex ligation probe amplification
PMS: Phelan-McDermid syndrome
SD: Standard deviation
SNP-array: Single-nucleotide polymorphism array
VOUS: Variant of uncertain significance
