# Supplementary material for: A Brazilian cohort of individuals with Phelan-McDermid syndrome: genotype-phenotype correlation and identification of an atypical case
Source: J Neurodev Disord. 2019 Jul 18;11:13. doi: 10.1186/s11689-019-9273-1 (PMC6637483; doi:10.1186/s11689-019-9273-1)
Supplement: Supplementary file 1 — Figure S1. Multiplex ligation-dependent probe amplification (MLPA) test of atypical individual (P32) and his parents, confirm that its a de novo deletion. Figure S2. 22q13 deletions in 22 patients with renal abnormalities. Table S1. Frequency of dysmorphic features seen in PMS individuals. Table S2. Clinical findings in Brazilian PMS individuals. N/A = not available; + = comorbidity is present in the individual; − = comorbidity absent in the individual; F = female, M = male. Table S3. Clustering analysis of PMS individuals according to the sex and comorbidities seen among them. Table S4. p-value of Mann Whitney test performed for each comorbidity’s frequency and deletion size in our cohort (N = 34). Table S5. Fourteen individuals with 22q13.3 deletion and renal abnormalities previously described in the literature for which data was available online. Table S6. One hundred and one patients from three previously published works (Tabet et al, 2017; Lei et al, 2016; Soorya et al, 2013) and from our cohort included in the analysis of the frequency of renal abnormalities. All chromosomal coordinates are based on the hg19 version of the human reference genome. Color blue = individuals from Tabet et al (2017), green = individuals from Soorya et al (2013), orange = individuals from our cohort, and black = individuals from Lei et al (2016). (DOCX 400 kb) [file 11689_2019_9273_MOESM1_ESM.docx]

**Additional file 1**


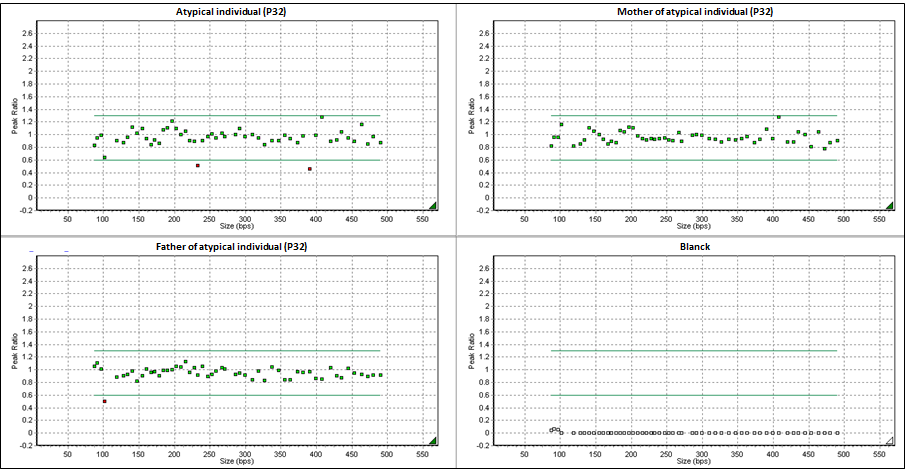


**Figure S1**. Multiplex ligation-dependent probe amplification (MLPA) test of atypical individual (P32) and his parents, confirm that its a de novo deletion.


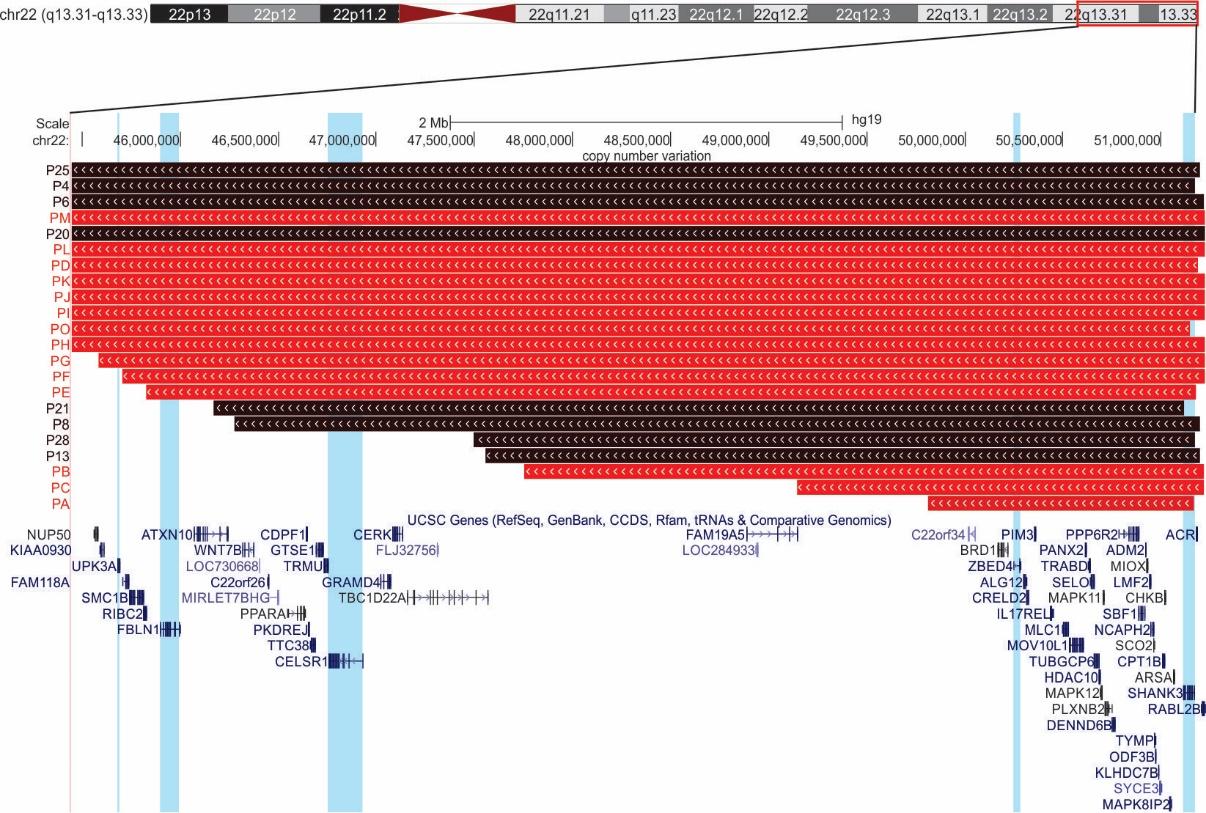


**Figure S2**. 22q13 deletions in 22 patients with renal abnormalities. Deletions found in our cohort are represented by black lines, those reported previously are shown in red (Tabet *et al*. 2017, Soorya *et al*. 2013, Lei *et al*. 2016). Candidate genes *FBLN1, CELSR1, ZBED4* and *SHANK3* gene are highlighted by the blue vertical lines.

Table S1. Frequency of dysmorphic features seen in PMS individuals.

| **Dysmorphic features** | **N** | **Total** | **%** |
| --- | --- | --- | --- |
| **Nails** | | | |
| Hyperconvex | 2 | 26 | 7.7% |
| Hypoplastic / Small | 2 | 26 | 7.7% |
| **Ears** | | | |
| Dysplastic Ears | 3 | 26 | 11.5% |
| Large ears (macrotia) | 7 | 26 | 26.9% |
| Ear low set | 2 | 26 | 7.7% |
| Prominent ears | 1 | 26 | 3.8% |
| Proeminent anthelix | 5 | 26 | 19.2% |
| Helix Prominent | 1 | 26 | 3.8% |
| **Eyes** | | | |
| Epicanthus | 2 | 26 | 7.7% |
| Ascending palpebral fissure | 3 | 26 | 11.5% |
| Descending palpebral fissure | 2 | 26 | 7.7% |
| Hypotelorism | 1 | 26 | 3.8% |
| Distichiasis | 16 | 26 | 61.5% |
| Strabismus | 3 | 26 | 11.5% |
| Long eyelashes | 3 | 26 | 11.5% |
| **Nose** | | | |
| Large/wide nose | 11 | 26 | 42.3% |
| Tubular nose | 4 | 26 | 15.4% |
| **Face** | | | |
| Flat face | 4 | 26 | 15.4% |
| Face long | 4 | 26 | 15.4% |
| Triangular face | 1 | 26 | 3.8% |
| Prominent mandible /prognathism | 1 | 26 | 3.8% |
| High forehead | 9 | 26 | 34.6% |
| **Philtrum** | | | |
| Long philtrum | 2 | 26 | 7.7% |
| Philtrum deep | 7 | 26 | 26.9% |
| Short philtrum | 1 | 26 | 3.8% |
| Wide philtrum | 2 | 26 | 7.7% |
| **Mouth** | | | |
| Down-turned mouth | 4 | 26 | 15.4% |
| Tented lip | 1 | 26 | 3.8% |
| Macrostomy | 2 | 26 | 7.7% |
| Thick lower lip | 2 | 26 | 7.7% |
| Thin upper lip | 1 | 26 | 3.8% |
| **Teeth** | | | |
| Small teeth | 1 | 26 | 3.8% |
| Spaced teeth | 1 | 26 | 3.8% |
| **Fingers** | | | |
| Clinodactyly | 15 | 26 | 57.7% |
| **Feet** | | | |
| Elongated feet | 1 | 26 | 3.8% |
| Foot broad | 3 | 26 | 11.5% |
| Overlapping toes | 4 | 26 | 15.4% |
| Short toes | 1 | 26 | 3.8% |
| Partial syndactyly of toes 2–3 | 2 | 26 | 7.7% |
| Toes widely spaced | 6 | 26 | 23.1% |
| Elongated toes | 3 | 26 | 11.5% |
| **Others** | | | |
| Sparce eyebrowns | 21 | 26 | 80.8% |

Table S2. Clinical findings in Brazilian PMS individuals. N/A = not available; + = comorbidity is present in the individual; - = comorbidity absent in the individual; F = female, M = male.

| **ID** | **Sex** | **Deletion size (Mb)** | **Lymphedema** | **Chewing difficulties** | **Hypohidrosis** | **Constipation and/or diarrhea** | **Sleep disturbance** | **Cardiac abnormalities** | **Recurring upper respiratory tract infections** | **Renal abnormalities** | **Strabismus** | **Gastroesophageal reflux** | **Hypotonia** | **Pain tolerance** | **Speech** |
| --- | --- | --- | --- | --- | --- | --- | --- | --- | --- | --- | --- | --- | --- | --- | --- |
| P1 | F | 2.4 | - | - | - | - | - | - | - | - | - | - | + | + | - |
| P2 | F | 7.8 | - | + | + | - | - | - | + | - | - | - | + | + | - |
| P3 | F | NA | - | + | - | + | + | - | - | - | + | + | + | N/A | - |
| P4 | M | 8.3 | N/A | + | - | + | + | - | - | + | + | N/A | + | + | - |
| P5 | M | NA | - | - | + | - | + | - | - | - | - | + | + | + | - |
| P6 | M | 8.3 | - | - | + | - | + | - | + | + | - | + | + | + | - |
| P7 | F | 1.5 | N/A | + | N/A | N/A | + | - | - | N/A | - | + | + | + | - |
| P8 | F | 4.9 | - | - | - | - | - | - | + | + | + | + | - | - | - |
| P9 | F | 2.4 | - | - | - | - | - | - | + | - | - | + | + | + | - |
| P10 | F | 2.7 | - | + | - | - | + | - | - | - | - | N/A | N/A | - | - |
| P11 | F | 0.5 | - | + | - | + | + | - | + | - | - | - | + | + | - |
| P12 | M | 49 | - | + | - | - | - | - | + | - | - | - | + | + | - |
| P13 | M | 3.6 | - | - | + | + | + | - | - | + | - | - | + | + | - |
| P14 | M | 0.9 | - | + | + | - | - | + | - | - | - | - | - | - | + |
| P15 | M | 7.7 | + | N/A | - | - | - | - | + | - | - | N/A | + | + | - |
| P16 | M | 5.9 | - | - | N/A | - | - | - | + | - | - | + | - | - | - |
| P17 | F | 74 | - | - | + | - | + | - | - | - | + | + | + | + | - |
| P18 | F | 0.1 | - | + | + | + | + | - | + | N/A | - | + | + | + | - |
| P19 | F | 1.5 | - | + | + | - | + | - | + | - | - | + | + | + | - |
| P20 | F | 8.0 | + | N/A | - | + | - | - | + | + | - | + | + | + | - |
| P21 | F | 4.9 | - | - | - | - | N/A | - | - | + | - | + | - | - | - |
| P22 | M | NA | N/A | - | - | - | - | - | - | - | - | + | - | - |  |
| P23 | F | NA | - | + | - | - | - | - | - | - | - | - | + | - | - |
| P24 | F | 7.9 | - | + | - | + | - | - | + | - | - | + | + | + | - |
| P25 | F | 8.79 | - | - | - | + | - | - | + | + | + | + | + | + | - |
| P26 | M | 2.1 | - | + | + | - | N/A | - | - | - | - | - | + | + | - |
| P27 | M | 9.0 | + | + | - | + | + | - | + | + | - | + | + | + | - |
| P28 | F | 3.67 | - | - | - | - | - | - | - | - | + | - | - | - | - |
| P29 | M | NA | N/A | - | + | + | - | - | - | + | - | + | + | - | - |
| P30 | M | 3.25 | - | - | N/A | + | - | + | - | - | N/A | - | + | + | + |
| P31 | M | 0.9 | - | + | - | + | - | - | - | - | - | - | + | + | - |
| P32 | M | 0.1 | - | + | - | + | - | - | + | - | - | - | N/A | - | + |
| P33 | F | 4.39 | + | + | N/A | N/A | - | - | - | - | N/A | + | + | + | - |
| P34 | M | 8.1 | - | - | - | - | + | - | - | - | N/A | - | N/A | - | - |

Table S3. Clustering analysis of PMS individuals according to the sex and comorbidities seen among them.

|  | **Group** | | | |
| --- | --- | --- | --- | --- |
|  | 1 | 2 | 3 | 4 |
| Female : Male | 6:4 | 6:3 | 4:0 | 0:6 |
| Deletion size (mean and range) | 4.28 Mb (0.07-8.18 Mb) | 5.2 Mb (0.1-9.05 Mb) | 5.58 Mb (3.67-8.79 Mb) | 1.22 Mb (0.04-3.25 Mb) |
| **Comorbidities** | | | | |
| Lymphedema | 0% (0/10) | 57% (4/7) | 0% (0/4) | 0% (0/6) |
| Chewing difficulty | 30% (3/10) | 100% (7/7) | 0% (0/4) | 83% (5/6) |
| Hypohidrosis | 56% (5/9) | 14% (1/7) | 0% (0/4) | 40% (2/3) |
| Constipation and/or diarrhea | 10% (1/10) | 86% (6/7) | 25% (1/4) | 50% (3/3) |
| Sleep disturbance | 60% (6/10) | 56% (5/9) | 0% (0/3) | 0% (0/5) |
| Cardiac abnormalities | 0% (0/10) | 0% (0/9) | 0% (0/4) | 33% (2/6) |
| Renal abnormalities | 20% (2/10) | 25% (2/8) | 100% (4/4) | 0% (0/6) |
| Recurring upper respiratory tract infections | 63% (5/8) | 86% (6/7) | 50% (2/4) | 33% (2/6) |
| Gastroesophageal reflux | 56% (5/9) | 86% (6/7) | 75% (3/4) | 0% (0/6) |
| Hypotonia | 88% (7/8) | 100% (9/9) | 25% (1/4) | 80% (4/5) |
| Increased pain tolerance | 88% (7/8) | 100% (9/9) | 25% (1/4) | 80% (4/5) |
| Speech | 0% (0/8) | 0% (0/7) | 0% (0/3) | 50% (3/6) |

| Table S4. p-value of Mann Whitney test performed for each comorbidity’s frequency and deletion size in our cohort (N=34) | |
| --- | --- |
| **Comorbidity** | **Mann-Whitney test (p-value)** |
| Increased pain tolerance | 0.850 |
| Hypotonia | 0.850 |
| Recurring upper respiratory tract infections | 0.367 |
| Gastroesophageal reflux | 0.106 |
| Sleep disturbance | 0.942 |
| Seizures (febrile and/or non-febrile) | 0.500 |
| Constipation and/or diarrhea | 0.577 |
| Renal abnormalities | **0.011** |
| Lymphedema | **0.048** |
| Strabismus | 0.374 |
| Cardiac abnormalities | 0.493 |
| Chewing difficult | 0.126 |
| Swallowing difficult | 0.842 |
| Hypohidrose | 0.215 |

| Table S5. Fourteen individuals with 22q13.3 deletion and renal abnormalities previously described in the literature for which data was available online. | | | | | | |
| --- | --- | --- | --- | --- | --- | --- |
| **Article** | **Individual ID (this article)** | **Individual ID (original article)** | **Deletion (hg19[GRCh37] - 22q13)** | | **Size (pb)** | **Phenotype** |
| Tabet et al (2017) | A | 27 | 49813066 | 51170164 | 1357098 | Hydronephrosis |
|  | B | 69 | 47751912 | 51219150 | 3467238 | Kidney abnormalities |
|  | C | 15 | 49145909 | 51219150 | 2073241 | Left kidney duplicity |
|  | D | 19 | 43759828 | 51186190 | 7426362 | Left pyeloureteral junction |
|  | E | 4 | 45828060 | 51178405 | 5350345 | Unilateral multicystic dysplastic kidney with normal renal function |
| Soorya et al (2013) | F | 28 | 45705241 | 51224208 | 5518967 | Not specified by patient, but authors report vesicoureteral reflux, hydronephrosis, renal agenesis, extrarenal pelvis, dysplastic kidney, and bilateral horseshoe kidneys and pyelectasis among the 12 patients with renal abnormalities. |
|  | G | 17 | 45583935 | 51224208 | 5640273 |  |
|  | H | 21 | 45428606 | 51224208 | 5795602 |  |
|  | I | 22 | 44800014 | 51224208 | 6424194 |  |
|  | J | 6 | 44427703 | 51224208 | 6796505 |  |
|  | K | 4 | 44321641 | 51224208 | 6902567 |  |
|  | L | 13 | 43745129 | 51224208 | 7479079 |  |
|  | M | 16 | 42918711 | 51224208 | 8305497 |  |
| Lei et al (2016) | O | P1 | 44882702 | 51146914 | 6264212 | Double kidney effusion |

| Table S6. One hundred and one patients from three previously published works (Tabet *et al*, 2017; Lei *et al*, 2016; Soorya *et al*, 2013) and from our cohort included in the analysis of the frequency of renal abnormalities. All chromosomal coordinates are based on the hg19 version of the human reference genome. Color blue = individuals from Tabet *et al* (2017), green = individuals from Soorya *et al* (2013), orange = individuals from our cohort, and black = individuals from Lei *et al* (2016). | | | | | | | |
| --- | --- | --- | --- | --- | --- | --- | --- |
| ID | Start | End | ZBED4 | CELSR1 | *UPK3A* | FBLN1 | |
|  |  |  | (hg19) 50247497-50283726 | (hg19) 46756731-46933067 | (hg19) 45680868-45691755 | (hg19) 45898719-45997014 | |
| individuals without renal abnormalities | | | | | | | |
| 5 | 45550500 | 51169045 | Yes | Yes | Yes | Yes | |
| 6 | 44187687 | 51169045 | Yes | Yes | Yes | Yes | |
| 8 | 43872933 | 51169045 | Yes | Yes | Yes | Yes | |
| 10 | 51072162 | 51169045 | No | No | No | No | |
| 11 | 50452208 | 51169045 | No | No | No | No | |
| 13 | 50883397 | 51178264 | No | No | No | No | |
| 14 | 47080299 | 51219009 | Yes | No | No | No | |
| 16 | 51123291 | 51219150 | No | No | No | No | |
| 18 | 51123291 | 51219150 | No | No | No | No | |
| 23 | 49513930 | 51218950 | Yes | No | No | No | |
| 26 | 49623769 | 51186190 | Yes | No | No | No | |
| 33 | 50937228 | 51178405 | No | No | No | No | |
| 34 | 51123291 | 51178405 | No | No | No | No | |
| 36 | 51123291 | 51219150 | No | No | No | No | |
| 37 | 51132603 | 51178405 | No | No | No | No | |
| 38 | 43331411 | 51178405 | Yes | Yes | Yes | Yes | |
| 39 | 43415599 | 51178264 | Yes | Yes | Yes | Yes | |
| 40 | 48802104 | 51178264 | Yes | No | No | No | |
| 41 | 44864865 | 51178264 | Yes | Yes | Yes | Yes | |
| 42 | 44934059 | 49522658 | Yes | Yes | Yes | Yes | |
| 47 | 46023639 | 51195728 | Yes | Yes | No | No | |
| 53 | 51116107 | 51170223 | No | No | No | No | |
| 55 | 43730323 | 51178264 | Yes | Yes | Yes | Yes | |
| 56 | 42320845 | 51178405 | Yes | Yes | Yes | Yes | |
| 57 | 42069936 | 51178264 | Yes | Yes | Yes | Yes | |
| 58 | 50055568 | 51178264 | Yes | No | No | No | |
| 59 | 43900960 | 49565875 | Yes | Yes | Yes | Yes | |
| 61 | 49995123 | 51178264 | Yes | No | No | No | |
| 64 | 50671279 | 51219150 | No | No | No | No | |
| 65 | 50004759 | 51178264 | Yes | No | No | No | |
| 66 | 49536180 | 51193680 | Yes | No | No | No | |
| 67 | 43057749 | 51219150 | Yes | Yes | Yes | Yes | |
| 68 | 48224328 | 51219150 | Yes | No | No | No | |
| 70 | 45795093 | 51304566 | Yes | Yes | No | No | |
| 71 | 45547030 | 51186390 | Yes | Yes | Yes | Yes | |
| 74 | 45522296 | 51170223 | Yes | Yes | Yes | Yes | |
| 76 | 51123491 | 51178264 | No | No | No | No | |
| 77 | 47648233 | 51211392 | Yes | No | No | No | |
| 78 | 49745407 | 51195728 | Yes | No | No | No | |
| 79 | 51121362 | 51234442 | No | No | No | No | |
| 80 | 48802104 | 51178264 | Yes | No | No | No | |
| 82 | 47357143 | 51178264 | Yes | No | No | No | |
| 83 | 50558914 | 51178264 | No | No | No | No | |
| 84 | 46204739 | 51178264 | Yes | Yes | No | No | |
| SH5 | 46143471 | 51224208 | Yes | Yes | No | No | |
| SH8 | 49574124 | 51224208 | Yes | No | No | No | |
| SH9 | 49028732 | 51224208 | Yes | No | No | No | |
| SH10 | 51122946 | 51224208 | No | No | No | No | |
| SH11 | 49028732 | 51224208 | Yes | No | No | No | |
| SH12 | 42773732 | 51224208 | Yes | Yes | Yes | Yes | |
| SH15 | 45902119 | 51224208 | Yes | Yes | No | No | |
| SH18 | 50077362 | 51224208 | Yes | No | No | No | |
| SH19 | 48551989 | 51206201 | Yes | No | No | No | |
| SH20 | 51083118 | 51224208 | No | No | No | No | |
| SH25 | 46787434 | 51224208 | Yes | Yes | No | No | |
| SH27 | 51115526 | 51234443 | No | No | No | No | |
| SH30 | 49004395 | 51224208 | Yes | No | No | No | |
| SH31 | 42822943 | 51224208 | Yes | Yes | Yes | Yes | |
| P1 | 48810119 | 51211393 | Yes | No | No | No | |
| P2 | 43371148 | 51186249 | Yes | Yes | Yes | Yes | |
| P7 | 49608334 | 51186249 | Yes | No | No | No | |
| P9 | 48771374 | 51171678 | Yes | No | No | No | |
| P10 | 48434307 | 51178213 | Yes | No | No | No | |
| P11 | 50667787 | 51171678 | No | No | No | No | |
| P12 | 51122360 | 51171678 | No | No | No | No | |
| P14 | 48627093 | 49525079 | Yes | No | No | No | |
| P15 | 43492638 | 51197766 | Yes | Yes | Yes | Yes | |
| P16 | 45235285 | 51171678 | Yes | Yes | Yes | Yes | |
| P17 | 51104247 | 51178574 | No | No | No | No | |
| P19 | 49595567 | 51178405 | Yes | No | No | No | |
| P24 | 49595567 | 51178405 | Yes | No | No | No | |
| P26 | 49123097 | 51224252 | Yes | No | No | No | |
| P27 | 42152988 | 51211392 | Yes | Yes | Yes | Yes | |
| P30 | 47963467 | 49565875 | Yes | No | No | No | |
| P31 | 50274217 | 51197766 | Yes | No | No | No | |
| P32 | 51112491 | 51224252 | No | No | No | No | |
| P33 | 46814671 | 51211392 | Yes | Yes | No | No | |
| P34 | 43094876 | 51197838 | Yes | Yes | Yes | Yes | |
| Individuals with renal abnormalities | | | | | | | |
| **4** | **45828060** | **51178405** | **Yes** | **Yes** | **No** | | **No** |
| **15** | **49145909** | **51219150** | **Yes** | **No** | **No** | | **No** |
| **19** | **43759828** | **51186190** | **Yes** | **Yes** | **Yes** | | **Yes** |
| **27** | **49813066** | **51170164** | **Yes** | **No** | **No** | | **No** |
| **69** | **47751912** | **51219150** | **Yes** | **No** | **No** | | **No** |
| **L1** | **44882702** | **51146914** | **Yes** | **Yes** | **Yes** | | **Yes** |
| **SH4** | **44321641** | **51224208** | **Yes** | **Yes** | **Yes** | | **Yes** |
| **SH6** | **44427703** | **51224208** | **Yes** | **Yes** | **Yes** | | **Yes** |
| **SH13** | **43745129** | **51224208** | **Yes** | **Yes** | **Yes** | | **Yes** |
| **SH16** | **42918711** | **51224208** | **Yes** | **Yes** | **Yes** | | **Yes** |
| **SH17** | **45583935** | **51224208** | **Yes** | **Yes** | **Yes** | | **Yes** |
| **SH21** | **45428606** | **51224208** | **Yes** | **Yes** | **Yes** | | **Yes** |
| **SH22** | **44800014** | **51224208** | **Yes** | **Yes** | **Yes** | | **Yes** |
| **SH28** | **45705241** | **51224208** | **Yes** | **Yes** | **No** | | **No** |
| **P4** | **42865291** | **51219009** | **Yes** | **Yes** | **Yes** | | **Yes** |
| **P6** | **41194910** | **49397713** | **Yes** | **Yes** | **Yes** | | **Yes** |
| **P8** | **46276401** | **51197766** | **Yes** | **Yes** | **No** | | **No** |
| **P13** | **47557877** | **51197766** | **Yes** | **No** | **No** | | **No** |
| **P20** | **43213659** | **51224252** | **Yes** | **Yes** | **Yes** | | **Yes** |
| **P21** | **46168628** | **51115526** | **Yes** | **Yes** | **No** | | **No** |
| **P25** | **42399686** | **51197766** | **Yes** | **Yes** | **Yes** | | **Yes** |
| **P28** | **47497833** | **51171678** | **Yes** | **No** | **No** | | **No** |
| **1** | **44882702** | **51146914** | **Yes** | **Yes** | **Yes** | | **Yes** |
